# Insights into Pathology and Pathogenesis of Coronavirus Disease 2019 from a Histopathological and Immunological Perspective

**DOI:** 10.31662/jmaj.2021-0041

**Published:** 2021-07-13

**Authors:** Shun Iida, Takeshi Arashiro, Tadaki Suzuki

**Affiliations:** 1 Department of Pathology, National Institute of Infectious Diseases, Tokyo, Japan

**Keywords:** COVID-19, SARS-CoV-2, Pathology, Immunity, Pathogenesis

## Abstract

Since the first case of COVID-19 was reported in Wuhan, China, in December 2019, the SARS-CoV-2 epidemic has spread all over the world and has become a significant public health issue. The development of treatments for COVID-19 is currently in progress; however, their effects remain limited, and the development of more effective therapeutics is desired. Thus, sufficient understanding of the pathophysiology of COVID-19 is essential to develop effective therapeutics for this disease. Pathological analyses in particular play an important role to demonstrate the causal link between an infectious disease and the pathogen and elucidate the mechanism of pathogenesis. As per pathological analyses to date, respiratory organs are identified as the major affected organs in most COVID-19 cases; also, various lesions were noted in other organs. Further, there have been increasing reports that show that the immune responses of the host contribute to the deterioration of the pathological condition of COVID-19, and a novel concept of MIS-C/MIS-A is also being established. Thus, in this article, we have provided an overview of the pathology of COVID-19 from a histopathological and immunological perspective focusing on the mechanisms of COVID-19 pathogenesis.

## Introduction

Coronavirus disease 2019 (COVID-19) is an emerging infectious disease caused by severe acute respiratory syndrome coronavirus 2 (SARS-CoV-2) ^(1)^. Since the first case of “pneumonia of unknown etiology” was reported in Wuhan, China, in December 2019 ^(2)^, COVID-19 has rapidly spread internationally. More than 100 million infected persons have been reported in 192 countries and regions as of March 2021 ^(3)^. The number of deaths has reached over 2.5 million ^(3)^, thus making the disease a significant global public health emergency. In Japan, the first confirmed case of COVID-19 was reported in January 2020. The cumulative number of infected persons has already reached over 400,000, and the number of deaths has exceeded 8,000 ^(4)^. Development of effective therapeutics and vaccines for an infectious disease is important to control the infection. Although candidate therapeutics for COVID-19 have mainly been searched among existing drugs since the beginning of the outbreak, their effects remain limited; thus, developing more effective therapeutics is required.

Understanding the pathogenesis of emerging infectious diseases can be a valuable contribution to the development of effective therapeutics and vaccines for these diseases. It is particularly important to clarify the relationship between the disease and the pathogen by elucidating the pathogen’s distribution in organs/tissues and to understand the pathogenic mechanism by analyzing the morphological changes in organs/tissues. However, it is impossible to obtain detailed information on where the pathogen is localized in the body and the pathology it causes in tissues based solely on conventional pathogen testing or diagnostic imaging such as X-rays, computed tomography (CT) scans, and magnetic resonance imaging (MRI). Pathological analysis using tissue samples of patients is therefore essential to understand the pathogenesis of COVID-19. In addition, it has gradually become apparent that the host immune response plays an important role in the pathogenesis of COVID-19. Thus, it is considered essential to examine the interaction between SARS-CoV-2 and host immunity to elucidate the pathophysiology of COVID-19.

This article outlines the pathological characteristics of COVID-19 on the respiratory and other organs, which have mainly been demonstrated by autopsies. We also describe an outline of the current understanding of the immunological response in COVID-19.

## Pathology of COVID-19

### Pathology of COVID-19 pneumonia

The most common pathology of COVID-19 is pneumonia. Severe pneumonia may be accompanied by acute respiratory distress syndrome (ARDS), which can be fatal in some cases. The main host receptor for SARS-CoV-2 is angiotensin-converting enzyme 2 (ACE2). ACE2 is mainly expressed on the surface of airway epithelial cells including type II alveolar epithelium. It has been considered that SARS-CoV-2 binds to ACE2 via the receptor-binding domain (RBD) in the S1 domain of the spike protein to infect airway epithelial cells ^(5)^.

**Figure 1.**
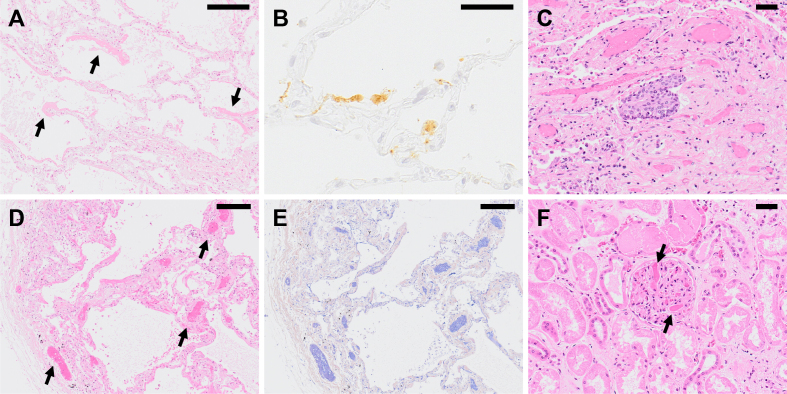
Histopathological findings of COVID-19 pneumonia. (A) A lung specimen of COVID-19 pneumonia obtained from an autopsied case showing exudative phase of DAD with formation of hyaline membrane (arrow) along alveolar septa. Hematoxylin & eosin (H&E) staining. Scale bar indicates 200 μm. (B) Detection of SARS-CoV-2 antigen in COVID-19 pneumonia by immunohistochemistry using a rabbit polyclonal antibody against SARS-CoV-2 antigens ^(65)^. Positive signals (brown) are observed on alveolar epithelial cell and alveolar macrophages. Scale bar indicates 50 μm. Panels A and B are from the same case at the same time. (C) A lung specimen of COVID-19 pneumonia showing organizing phase of DAD with squamous cell metaplasia. H&E staining. Scale bar indicates 50 μm. (D) Exudative phase of DAD accompanied with prominent microvascular thrombosis (arrow) observed in COVID-19 pneumonia. H&E staining. Scale bar indicates 200 μm. (E) Microthrombi (blue) are highlighted by phosphotungstic acid hematoxylin (PTAH) staining on the same specimen as panel D. Scale bar indicates 200 μm. (F) Microthrombi formation (arrow) in a glomerulus of the kidney from a COVID-19 autopsy case. H&E staining. Scale bar indicates 50 μm.

COVID-19 pneumonia is characterized by the finding of diffuse alveolar damage (DAD), which is the histopathological pattern representing ARDS ^(6)^. In general, the histopathological pattern of DAD changes over time from exudative to organizing, and fibrotic stage, which is also true in COVID-19. The histopathological pattern in early stage of COVID-19 pneumonia onset is exudative pattern; thereafter, organizing and fibrotic patterns tend to develop over time after the onset ^(7)^. The major characteristics of COVID-19 pneumonia is that lesions in the different time phases are found simultaneously in close proximity to each other within the lung lobe, and an autopsy on the lung tissue often shows various DAD lesions on the different stages, from exudative to fibrotic stage, in a pulmonary lobe. Based on these findings, it is suggested that SARS-CoV-2 does not infect all alveoli of the lung simultaneously, but that the virus infection gradually spreads throughout the lung tissues to form extensive pulmonary pathology, which eventually leads to respiratory failure. The representative pathological findings of COVID-19 pneumonia are as follows (Figure 1):

(1) Exudative stage: Characteristic formation of a hyaline membrane along the alveolar wall, with inflammatory cell infiltration of the alveolar wall. At this stage, no obstruction of the alveolar space due to organizing exudate or fibrosis of the interstitium is observed.

(2) Organizing stage: As the lesion progresses, organized hyaline membrane can be observed. The alveolar space becomes filled with organized exudate, and macrophage infiltration is observed. Detachment of alveolar epithelial cells, squamous cell metaplasia, and type II alveolar epithelial cell hyperplasia are noted, while the alveolar walls show fibroblast proliferation and inflammatory cell, mainly plasma cell, infiltration. Findings like congestion and hemorrhage in the alveolar space may also be present.

(3) Fibrotic stage: If the lesion worsens further, proliferation of fibroblasts in the interstitium and collagen fiber deposits lead to obstruction of the alveolar space and modification of the alveolar structure.

Immunofluorescence and immunohistochemistry analyses using anti-SARS-CoV-2 antibody revealed positive signals for viral antigens in the alveolar epithelium, suggesting that SARS-CoV-2 invades the alveolar epithelium ^(6)^. Notably, alveolar epithelium in regions lacking inflammatory cell infiltration or hyaline membrane formation, and showing almost no histopathological changes, often is positive for viral antigen, suggesting that SARS-CoV-2 infection into the alveolar epithelium precedes the host immune response, which is responsible for the formation of histopathological changes in COVID-19. In addition, alveolar macrophages were also positive for viral antigen, but it has not been clarified whether SARS-CoV-2 replicates in the macrophages or the macrophages just phagocytose the SARS-CoV-2-infected alveolar epitheliums or the virions.

### Pathological features of COVID-19 other than pneumonia

Detailed clinical and pathological analyses have revealed that COVID-19 also damages the cardiovascular system, kidneys, gastrointestinal system, and central nervous system (CNS) among others ^(8)^. It has been reported that SARS-CoV-2 is also detected in organs other than the respiratory tract, but some researchers pointed out that intracellular structures are misidentified as viral particles in some reports ^(9)^. It has also been found that the host immune response damages various organs, and this will be described later. It is therefore considered necessary to further investigate whether such pathology in non-respiratory organs is the result of direct insult caused by SARS-CoV-2 infection, or whether it is due to an indirect effect such as host immune-mediated mechanism. Although the definitive conclusion at this time is that COVID-19 is a respiratory viral infection that primarily targets the respiratory organs, there are various pathological conditions other than pneumonia that have been associated with COVID-19. Here, we discuss thrombosis, renal dysfunction, cardiovascular disorder, and neurological manifestation of COVID-19. We also expound on the vertical transmission potential of SARS-CoV-2, focusing on placental pathology.

### COVID-19 thrombosis

Thrombosis is identified as one of the relatively frequent complications of severe COVID-19. A report from the United States describes that microthrombi were observed in the lungs of 5 of 14 patients with COVID-19 during autopsy, and thrombi were also observed in the kidney of 1 of them ^(10)^. Further, as per autopsy findings, all nine examined cases in the United Kingdom indicated the presence of thrombi in the organs as follows: lungs in eight cases (89%), heart in five cases (56%), and kidney in four cases (44%) ^(11)^. As described above, COVID-19 can cause thrombosis in various organs, and it has been reported that the frequency of thrombus formation is especially high in the lung.

In a study that compares the pathological findings of pulmonary thrombosis between lung tissue obtained during autopsy of COVID-19 with that of ARDS secondary to influenza A (H1N1pdm09), a pattern of DAD was observed in both, but endothelial injury and microangiopathy with widespread thrombosis were only found in individuals infected with COVID-19, suggesting a relationship between thrombosis and endothelial injury with COVID-19 ^(12)^. It has been pointed out that viral infection of endothelial cells may cause endothelial injury since ACE2, the receptor of SARS-CoV-2, is expressed in the vascular endothelium ^(13)^. However, there are conflicting reports on the direct viral infection of vascular endothelial cells ^(9)^, and this remains inconclusive.

### COVID-19 renal dysfunction

Renal dysfunction is also one of the relatively frequent complications of severe cases of COVID-19. The incidence of acute kidney injury (AKI) in patients with COVID-19 has been reported to be 5-15% in China ^(14)^ and 37% of inpatients in the United States ^(15)^. The mortality rate of concurrent AKI has reached 60-90% ^(14)^, with a poor prognosis.

According to a report from China which analyzed pathological findings of the kidneys of autopsied COVID-19 patients, varying degrees of findings suggesting proximal tubular injury such as disappearance of the brush border of the proximal tubular epithelium, vacuolar degeneration, necrosis, and dilation of the proximal tubule were observed ^(16)^. Other pathological findings include renal tubular epithelial cell casts and glomerular microthrombi. No findings of vasculitis or interstitial nephritis were noted ^(16)^. ACE2 is expressed in the proximal tubular epithelium, and immunofluorescence-positive signals of viral antigen were found in proximal tubular epithelium cells ^(16)^, suggesting that the kidney may have been infected with SARS-CoV-2. However, there have only been a few reports of isolation of the virus from urine to date ^(17)^, and direct infection of the urinary system has not been definitively confirmed.

### Cardiovascular manifestation of COVID-19

Although exact mechanism of cardiac injury remains unknown, several studies have found that cardiac injury as measured by troponin elevation were associated with a significantly higher mortality among patients hospitalized with COVID-19 ^(18)^. One potential cause of cardiac injury is direct insult from the virus. Some studies report the presence of SARS-CoV-2 antigen or RNA in the heart tissues of autopsied cases ^(19), (20)^. However, the detection frequency is unknown, and no convincing evidence as to whether live viral particle can indeed invade the heart as viremia (note that it is the presence of infectious virus in blood, not viral RNA) has not been reported and is expected to be extremely rare. As SARS-CoV-2 infection can cause systemic inflammation, potential to trigger myocarditis was a concern as seen in some other viruses. In several studies, MRI was utilized in an attempt to identify myocarditis among recovering COVID-19 cases, but the results remain conflicting ^(21), (22), (23)^. In a comprehensive literature review of autopsy reports with a total of 277 hearts, 20 cases (7.2%) were considered to have myocarditis by the authors in the original reports, but the diagnosis of myocarditis in 16 of these cases were deemed questionable by authors of the literature review ^(24)^. Therefore, the true incidence of myocarditis is considered to be quite low. In the same literature review with 277 cases, authors have also evaluated the frequency of acute, potentially COVID-19-related cardiovascular histopathologic finding such as macro- or microvascular thrombi, inflammation, or intraluminal megakaryocytes ^(24)^. As per their findings, it was determined that 47.8% of cases had potentially COVID-19-related cardiovascular histopathologic finding in the autopsied hearts. These findings may reflect cardiac injury observed in COVID-19 patients.

With regard to vascular pathology, some researchers hypothesize that the main pathological manifestation of COVID-19 is systemic endothelial injury, dysfunction, and inflammation (endotheliopathy) ^(12), (13)^. This is supported by studies reporting the presence of SARS-CoV-2 antigen or RNA in endothelium, suggesting direct infection in the vascular endothelium ^(13), (19), (25)^. It is most certain that COVID-19 can cause thromboembolism in blood vessels of various sizes affecting these endothelium or organs receiving blood supply through these vessels ^(26), (27)^, but further studies are necessary to fully understand whether peripheral vascular injury including endotheliopathy are truly one of the hallmarks of COVID-19.

### Neurological manifestation of COVID-19

With the increasing number of cases, neurological manifestations of COVID-19 such as headaches, dizziness, hyposmia, and dysgeusia have been largely recognized. These symptoms seem to result from various mechanisms such as virus-induced hyperinflammatory and hypercoagulable states, direct virus infection of the CNS, and post-infectious immune-mediated processes ^(28)^. However, detailed mechanisms of COVID-19-associated complications in nervous systems are not fully understood yet.

At present, several post-mortem studies have been conducted to investigate the pathological changes in COVID-19 cases, reporting various histopathological findings in the CNS ^(29), (30)^. An autopsy case series was carried out in the Netherlands, evaluating 21 autopsied brains. This study revealed two notable histopathological findings in the brains of COVID-19 cases: extensive inflammation in the olfactory bulbs and formation of microglia nodule in the medulla oblongata ^(19)^. Olfactory bulb and medulla oblongata play pivotal role in olfactory and gustatory sensation, respectively. However, association between those histopathological findings and hyposmia or dysgeusia is still unknown. Another post-mortem case series study in Germany examined autopsied brain tissues of COVID-19 cases revealing that SARS-CoV-2 RNA and proteins were detected by PCR and immunohistochemistry, respectively, in 21 out of 40 (53%) autopsied cases ^(31)^. They also reported some remarkable histopathological findings such as activation of microglia and infiltration of CD8^+^ cytotoxic T cells, affecting perivascular and parenchymal area in the brainstem and cerebellum. It is noteworthy that the severity of these changes was not associated with the distribution of SARS-CoV-2 ^(31)^. These findings suggest that CNS lesions can be developed through immunological mechanism rather than direct injury by SARS-CoV-2 infection. In contrast to these two studies, encephalitis or other specific changes related to virus infection were not observed in 18 autopsy cases of COVID-19 in the United States ^(32)^.

Taken altogether, widespread inflammation in the CNS may be a pathognomonic finding of COVID-19 and could be one of the mechanisms underlying neurological symptoms, but further studies are needed to elucidate the exact mechanism.

In addition, it is important to understand that whether SARS-CoV-2 directly infects the nervous system is still controversial since PCR and immunohistochemistry can develop false-positive results caused by detection of virus RNA in the blood or low specificity of antibody ^(32)^.

### Vertical transmission potential of SARS-CoV-2 with a focus on transplacental transmission and placental pathology

Vertical transmission can be classified into *in utero* (e.g., transplacental transmission), peripartum (e.g., transvaginal transmission during birth), or postnatal (e.g., transmission from breastfeeding). Since the early stage of the pandemic, the vertical transmission potential of SARS-CoV-2 has been a serious concern ^(33)^. However, accumulating evidence suggests that vertical transmission from pregnant women with SARS-CoV-2 infection is possible, but rare (2-3%, depending on reports) ^(34), (35), (36)^. Specifically, regarding transplacental transmission, SARS-CoV-2 antigen or RNA in placental tissue has also been limited with only a few reported suspected transplacental transmission ^(37), (38), (39), (40), (41)^. Structures considered to be SARS-CoV-2 viral particles in placental tissue using electron microscopy have been reported ^(37), (42)^, but the interpretation is complex ^(9)^. Also, it is difficult to evaluate whether pathological abnormalities observed is due to SARS-CoV-2 infection. One study examined placentas from 44 women with SARS-CoV-2 infection and 44 without infection as controls ^(41)^. The researchers found no statistical significance in maternal vascular malperfusion between infected individuals (16/44) and uninfected individuals (8/44), which may be due to small sample size. They noted that the odds of maternal vascular malperfusion lesions increased significantly with disease severity (odds ratio 2.09 [95%CI, 1.11-3.97]). The possible reason behind apparently infrequent vertical transmission through placenta or breastfeeding is rarity of viremia (note that it is the presence of infectious virus in the blood, not viral RNA). Scarce transplacental transmission is also supported by reduced placental expression of ACE2 and the serine protease transmembrane serine protease 2 (TMPRSS2) ^(38), (41)^, both of which are required for SARS-CoV-2 to enter into host cells ^(43)^.

## Immunity of COVID-19

### Immune response to SARS-CoV-2

The immune response to SARS-CoV-2 is currently being examined from various perspectives. Here, we give an overview of humoral immunity and cell-mediated immunity against SARS-CoV-2. Antibodies are identified to play a central role in humoral immunity. As with other infectious diseases, it has been shown that SARS-CoV-2 infection induces the production of antibodies. Serum IgM and IgG become positive in 1 to 2 weeks after onset of disease in many patients infected with SARS-CoV-2 ^(44)^. Although there is a report of IgA induction against the spike within 1 week after the onset of disease, prior to IgM and IgG production ^(45)^, it requires further verification because such a finding differs from the normal immunoglobulin kinetics. A decrease in serum antibody titer over time has mainly been reported for asymptomatic and mild patients ^(46)^, but similar phenomena have been reported with SARS-CoV and HCoV-229E, a seasonal coronavirus, and this finding is therefore not considered a phenomenon specific only to SARS-CoV-2. The association between decreased antibody titer and loss of protection against SARS-CoV-2 infection (i.e., correlate of protection) have not been elucidated, and long-term follow-up studies are needed to understand this.

A study that analyzed the serum of COVID-19 patients in the recovery phase has shown that antibodies induced by SARS-CoV-2 infection are mainly against spike protein (S-antigen) and nucleocapsid protein (N-antigen) as antigens. Antibodies to ORF8, ORF9b, and NSP5 are also shown to be induced ^(47)^. Among these, neutralizing antibodies against SARS-CoV-2 are mainly considered to recognize S-antigen ^(48)^. Interestingly, while a positive correlation between the severity of COVID-19 and the neutralizing antibody titer has been indicated ^(49)^, there is also a report denying a significant relationship between them ^(50)^.

Findings suggesting the importance of cell-mediated immunity have also been accumulated. Studies showing decreased CD4^+^ T cells and CD8^+^ T cells in the peripheral blood of patients with severe COVID-19 ^(51), (52)^, and those suggesting a correlation between the severity of COVID-19 and T-cell response, have been reported ^(53), (54)^. For cell-mediated immunity, more diverse viral antigens are targeted compared to humoral immunity, and the immune responses to NSP3, NSP4, ORF8, etc., besides S-antigen and N-antigen, have been confirmed ^(55)^.

Currently, measurement of the antibody titer for binding to S-antigen using enzyme-linked immunosorbent assay (ELISA) and assessment of the neutralizing antibody titer in the neutralization assay are often used to verify the efficacy of a vaccine. However, no definitive immunological surrogate marker that correlates with the prevention of infection/onset of SARS-CoV-2 has been demonstrated ^(56)^. It is not only important to elucidate the mechanism of protection against SARS-CoV-2, but also to explore the immunological surrogate markers from the viewpoint of vaccine development.

### Relationship between the pathogenesis of COVID-19 and immunity

The relationship between SARS-CoV-2 and immunity has been described here mainly from the viewpoint of protection against infection. It has been pointed out that immunity itself may be involved in the enhanced severity of COVID-19.

Since the early stage of the epidemic of SARS-CoV-2, pediatric patients with COVID-19 presenting with symptoms similar to those of Kawasaki disease have been reported mainly in Europe and the United States ^(57), (58), (59)^. Although the symptoms are relatively diverse, non-respiratory symptoms such as cardiac disorder, shock, and abdominal pain are deemed characteristic of the disease. Blood tests showed increased inflammatory markers such as C-reactive protein (CRP). As reports have accumulated, it has gradually become clear that systemic inflammation is an essential element of this disease, and this condition has been called multisystem inflammatory syndrome in children (MIS-C) ^(60)^. Recently, a similar condition has been noted to occur in adults too, referred to as multisystem inflammatory syndrome in adults (MIS-A) ^(61)^. Although there are still many unresolved points about the pathological basis of MIS-C and MIS-A, a theory of an excessive immune response due to delayed elimination of the virus from the body and a prolonged interferon response has been proposed ^(60)^. Importantly, the organ damage seen with MIS-C and MIS-A is not caused by viral infection into that organ, but the pathological condition is caused by a systemic inflammatory response.

The concept called antibody-dependent enhancement (ADE) is also important in the immunopathology ^(62)^. ADE is identified as a phenomenon in which antibodies against viral antigens cause worsening of the viral infection disease state as the name implies. The mechanism of development of ADE is roughly classified into two types. One is that virus-bound antibodies bind to Fc receptors to promote viral uptake by monocytes and macrophages and intracellular viral proliferation. Another is that immune complexes formed by the binding of antibody to the virus induce the recruitment of immunocompetent cells, promotion of secretion of inflammatory cytokines, and activation of complement, inducing excessive immune responses. Antibodies against viral antigens worsen the state of infectious disease in both cases. Since the latter does not involve virus proliferation, it has been pointed out that vaccination may cause ADE. It is also known that disease progression accompanied by eosinophil infiltration called enhanced respiratory disease (ERD) occurs due to re-infection after recovery from the first infection or infection after vaccination in animal models infected with SARS-CoV or MERS-CoV; thus, vaccine-associated enhanced disease (VAED) such as vaccination-related ADE and ERD are considered the largest barriers to the development of these coronavirus vaccines ^(63), (64)^. It is presently unknown if SARS-CoV-2 vaccination may cause VAED or not. For the development of SARS-CoV-2 vaccines, it is necessary to pay sufficient attention to the occurrence of these events and to conduct additional verification in animal models.

## Conclusions

COVID-19, which started as an outbreak in Wuhan, China, in December 2019 was initially reported as “pneumonia of unknown origin.” The subsequent studies revealed that SARS-CoV-2 is the causative agent. As a huge amount of study has been conducted, it has been clarified that COVID-19 patients present with various pathological conditions in various organs throughout the body including thrombosis, renal dysfunction, cardiovascular complications, and neurological dysfunction in addition to pneumonia, and that the host immune response causes damage to various organs. However, there are still many unclear points about the mechanism of development of these pathological conditions. There is controversy over whether or not the SARS-CoV-2 is directly involved, especially regarding pathological conditions other than pneumonia.

Pathological analyses have played a major role in elucidating the pathogenesis of COVID-19, and autopsies have provided many valuable insights. Furthermore, immunological analyses have not only started to clarify the host’s protective mechanism against SARS-CoV-2, but it also helped to understand the pathogenic mechanism of COVID-19. We hope that the insight into the mechanism of COVID-19 pathogenesis from the viewpoints of pathology and immunology will lead to the development of effective therapeutics.

## Article Information

### Conflicts of Interest

None

### Sources of Funding

The work was supported in part by the Emerging/Re-emerging Infectious Diseases Project of Japan, from the Japan Agency for Medical Research and Development, AMED under Grant Number JP21fk0108104.

### Acknowledgement

We thank Ms. Yuko Sato (National Institute of Infectious Diseases) for preparation of H&E staining and IHC slides of COVID-19 lung tissue sections.

### Author Contributions

SI, TA, and TS wrote the original draft of the manuscript and contributed to the review and editing of the manuscript.
